# Clinical efficacy and safety of tirofiban combined with conventional dual antiplatelet therapy in ACS patients undergoing PCI

**DOI:** 10.1038/s41598-021-96606-y

**Published:** 2021-08-25

**Authors:** Yong-zhe Guo, Zi-wen Zhao, Shu-mei Li, Liang-long Chen

**Affiliations:** Department of Cardiology, Fujian Medical University Union Hospital, 29 Xin-Quan Road, Fuzhou, 350001 Fujian China

**Keywords:** Drug safety, Cardiology, Diseases

## Abstract

Challenges remain for clinicians over balancing the efficacy of active antithrombotic therapy and simultaneous bleeding reduction in patients. The clinical data of 347 patients with acute coronary syndrome (ACS) undergoing percutaneous coronary intervention (PCI) were retrospectively analyzed. On the basis of the given tirofiban, the patients were assigned into three different dose groups: high dose group (group H), medium dose group (group M), and low dose group (group L). The tirofiban efficacy was evaluated in terms of major adverse cardiovascular event (MACE) parameters and lab endpoints, including platelet count and function. The tirofiban safety was assessed by the occurrence of bleeding events. The patients were followed up for 1 month after the PCI. No significant difference in MACE events was evident among these groups (*p* > 0.05). Groups H and M reported an obvious reduction in platelet count (*p* < 0.05 for both) and an increased platelet inhibition rate (*p* < 0.05 for both). Group H showed a higher rate of total bleeding events than the other groups (Group H vs. Group M: 34.4% vs. 16.5%; Group H vs. Group L: 34.4% vs. 10.3%; *p* < 0.05 for both). A proper administration of a low dose of tirofiban may be a superior alternative in treating ACS patients, which can produce a similar favorable clinical outcome and a decrease in bleeding complication.

## Introduction

Acute coronary syndrome (ACS) is an increasingly prevalent condition in the elderly population worldwide^1^. As secondary changes to unstable atheromatous plaque, it is often characterized by superficial platelet aggregation, platelet shedding, and other phenomena, which trigger the serial events of a sharp decrease in regional myocardial blood flow perfusion, an aggravation of myocardial ischemia, and the formation of platelet thrombus^2^. The activation of platelets and the coagulation system can directly influence the clinical results^3,4^, such as patient's cardiac function and long-term prognosis^5^.

Platelet agglutination not only impacts coronary thrombus but also induces microcirculatory dysfunction and vascular inflammation^6–8^. Therefore, anti-platelet aggregation is the basis for the ACS treatment of and a primary strategy for a successful operation. Although clinical practice guidelines in both the United States and Europe favor more potent, next-generation P2Y12 inhibitors, such as ticagrelor and prasugrel^9^, other alternative approaches such as clopidogrel are recommended for exceptions, including urgent operation needs, anticoagulation for atrial fibrillation, fibrinolytic therapy or the high bleeding risks such as thrombocytopenia, liver disease, or renal disease. Given this background, traditional antiplatelet strategies (aspirin combination with clopidogrel) will be our choice, which has proven beneficial for patients presenting with acute coronary syndromes in the CURE trial^10^. As one of the most powerful intravenous antiplatelet drugs, GP IIb/IIIa inhibitor (GPI) plays a pivotal role in inhibiting the final pathway of platelet aggregation. Facing high thrombus load or any other situations necessary for strengthening antiplatelet during operation, conventional double-antiplatelet therapy does not provide a satisfactory anti-platelet effect. The application of tirofiban may reduce peri-operative complications and quickly increase blood drug level and tissue perfusion^11^. Available research documents that the administration of tirofiban may improve the efficacy of clinical treatments with a lower all-cause mortality and a decreased risk of serious bleeding during peri-operative intervention^12^. Tirofiban has also been recommended as a bail-out treatment for coronary intervention therapy according to the established professional expert consensus^13–15^. However, challenges remain over balancing adequate antiplatelet therapy and bleeding control. To our best knowledge, little literature is available to shed light on the effective dose of Tirofiban in large-scale medical records^16,17^.

## Methods

A short-term, single-center, retrospective study was designed to evaluate the efficacy and safety of a multi-dose tirofiban treatment for 3,871 consecutive ACS patients from July 2015 to September 2019. The patients were examined for acute ST-segment elevation myocardial infarction (STEMI), non-acute ST-segment elevation myocardial infarction (NSTEMI), and unstable angina pectoris (UA). The inclusion criteria were as follows: (1) an age range of 18–80 years; (2) evidence of recurrent stenocardia; (3) signs of electrocardiographic ST-T deflection; and (4) a confirmed correlation of responsible lesion with clinical symptoms by coronary angiography. A total of 436 ACS patients, who had received PCI and intravenous tirofiban, met the inclusion criteria, from which 89 cases were excluded due to various reasons: 15 lacking angiography-verified pathological lesions, 37 undergoing balloon dilatation only, 19 requiring surgical bypass surgery due to left main vessel disease or multi-segmental lesions of multiple coronary arteries, 8 without a 1-month follow-up after the operation, and 10 presenting contraindications for the use of GPIs (active internal bleeding, known bleeding diathesis, intracerebral mass, or aneurysm, severe anemia and abnormal coagulation). The screening procedure left a total of 347 patients eligible for the retrospective analysis. Among the enrolled patients who had received tirofiban, the majority of them (94.5%) were treated with aspirin plus clopidogrel, of who 237 patients with atrial fibrillation (68.3%) received oral anticoagulant and 91 (26.2%) were treated with fibrinolytic therapy before interventional operation. This is consistent with the recommendations in the guidelines^15,18^ for patients who suffer atrial fibrillation and should be treated with oral anticoagulant and those receiving fibrinolytic therapy. According to the intravenous speed of tirofiban (per unit time), the patients were divided into the high-dose group (Group H, 0.10–0.15ug/kg/min, N = 61), the moderate-dose group (Group M, 0.05–0.10ug/kg/min, N = 218) and the low-dose group (Group L, < 0.05ug/kg/min, N = 68). Major medications were administered to all the patients according to the current clinical practice: aspirin (loading dose of 300 mg, then 100 mg qd), clopidogrel (loading dose of 300 mg, then 75 mg qd), ACEI/ARB, β-blocker, statin, and so on. These patients were unable to use potent P2Y12 because advanced age, coagulopathy, recent active gastrointestinal or cerebral hemorrhage, oral anticoagulant after atrial fibrillation or valve replacement surgery, or other factors ineligible for potent P2Y12.

PCI was performed by the transradial approach, using standard 6F or 7F guiding catheters. Unfractionated heparin was initiated at 100 IU/kg bolus and followed by the 12 U/kg/h infusion for any of the clinical indications, including atrial fibrillation, left ventricular thrombus or aneurysm, recent or recurrent venous thromboembolism, or deferred sheath removal. Coronary artery angiography (CAG) and PCI were performed by experienced practitioners in the center (designated as independent operation experience for more than 5 years). According to previous studies^19,20^, the administration of tirofiban [Grandpharma (China) Co., Ltd.; GYZ Zi H20041165] was divided into the following categories and the specific proportion was as follows: 29.7% for large, intraluminal residual thrombus, 24.2% for no (slow) reflow, 21.9% for threatening or acute vessel closure, 8.9% for complex lesions, and 15.3% for thrombotic complications. The dose recommendation was based on the judgment of the operator and was administered intravenously by continuous infusion for at least 48 h. Thromboelastography (TEG) was measured before and 48 h after the bolus tirofiban (ADP-induced turbidimetry by platelet aggregation instrument).

### End points and definitions

Primary clinical endpoint referred to major adverse cardiac events (MACEs), which consisted of total mortality, nonfatal myocardial infarction, nonfatal stroke, ischemia-driven target vessel revascularization and stent thrombosis. Nonfatal infarction was designated as the presence of: (1) recurrent ischemic chest pain lasting for more than 20 min; (2) reoccurrence of ST-segment deflection, T-wave inversion, or new pathognomonic Q waves in at least two contiguous leads; and (3) increase of cardiac troponin over the upper reference limit by at least 2 times. Stroke was defined as a new onset of focal or global neurological deficit for more than 24 h. Computed tomography was used to classify stroke as ischemic or hemorrhagic. According to the Academic Research Consortium^21^, a definite stent thrombosis was specified as an angiography or autopsy-confirmed thrombus that originates in the stent or in the segment 5 mm proximal or distal to the stent with the presence of acute coronary syndrome within a 48-h time window. Stent thrombosis contained acute and subacute stent thromboses, the former defined as within first 24 h after the intervention and the latter as between 24 h and 30 days after the intervention. The revascularization of the target vessel referred to the ischemia-driven percutaneous revascularization of the target vessel performed for restenosis or other complication.

Safety end point included bleeding events classified according to Thrombolysis in Myocardial Infarction (TIMI) criteria. Main bleeding complications were defined as follows: platelet reduction, referring to the postoperative platelet lower than 10*10^9/L; mild bleeding, referring to the postoperative hemoglobin decrease below 30–50 g/L, gross hematuria, petechia, gingival bleeding, common gastrointestinal hemorrhage or other phenomena; severe bleeding, to the hemoglobin decrease by more than 50 g/L, massive hemorrhage of digestive tract, or intracranial bleeding. Patients were followed-up for 30 days after the enrollment. The follow-up data were obtained by scheduled telephone interviews and out-patient visits. In addition to platelet count, TEG was also measured as the functional indicators of the effects of tirofiban^22^, which mainly comprised AA% (arachidonic acid inhibition rate), ADP% (adenosine diphosphate inhibition rate) and MA_ADP_ (ADP-induced platelet–fibrin clot strength) before and after the operation.

### Statistical treatment

According to previous literature^23^ and the requirements of statistical software, the minimum sample size of low-dose, medium-dose and high-dose groups was 65 cases, 210 cases and 60 cases, respectively. The data were analyzed with SPSS version 19 (IBM, Somers, NY, USA). This sample size allowed for an 80% statistical power to assess a significant difference (an error of 0.05). Continuous variables were presented as mean ± standard deviation and categorical variables as frequency and percentage. Analysis of variance (ANOVA) was performed to compare continuous variables in case of normally distributed value. Chi-square test was used to compare proportions and Fisher’s exact test was applied if the expected frequency was < 5. The Bonferroni method was used to calibrate the horizontal comparison of significance between groups in pairs. Multivariate logistic regression analysis was applied to analyze the potential factors correlated with the bleeding risks. The statistical significance was set at a two-sided probability value of < 0.05.

### Ethics declarations

All procedures performed in studies involving human participants were in accordance with the ethical standards of Ethics Committee of Fujian Medical University Union Hospital and with the 1964 Helsinki declaration and its later amendments or comparable ethical standards.

### Informed consent

Informed consent was obtained from all individual participants included in the study.

## Results

### Demographic features

The baseline demographic and clinical characteristics of the enrolled patients, who were treated with tirofiban during PCI, were shown in Table 1.

**Table 1:** Comparison of demographic and clinical features in the study groups.

	Group L (N = 68)	Group M (N = 218)	Group H (N = 61)	F/χ^2^	*P*
**Demographics**					
Age (years)	64.87 ± 10.41	65.52 ± 10.84	62.59 ± 8.19	1.927	0.147
Male, n (%)	52 (76.47)	179 (82.11)	54 (88.52)	3.184	0.204b
Smoker, n (%)	31 (45.59)	128 (58.72)	33 (54.10)	3.659	0.160b
Body-mass index (kg/m2)	25.52 ± 3.83	25.03 ± 3.36	24.50 ± 4.09	1.287	0.277
Duration time (hours)	52.85 ± 3.16	52.47 ± 3.67	52.32 ± 3.65	0.387	0.680
CYP2C19 gene					
Slow, n (%)	7 (10.29)	18 (8.26)	7 (11.48)	5.596	0.231b
Middle, n (%)	30 (44.12)	67 (30.73)	20 (32.79)		
Rapid, n (%)	31 (45.59)	133 (61.01)	34 (55.74)		
**Medical history**					
Gastrointestinal bleeding, n (%)	0 (0)	6 (2.75)	2 (3.28)	1.922	0.349a
Hematencephalon, n (%)	0 (0)	2 (0.92)	0 (0)	0.645	1.000a
Hypertension, n (%)	37 (55.41)	124 (56.88)	31 (50.82)	0.737	0.692b
Hyperlipidemia, n (%)	22 (32.35)	76 (34.86)	24 (39.34)	0.712	0.700b
Diabetes mellitus, n (%)	23 (33.82)	71 (32.57)	11 (18.03)	5.281	0.071b
Atrial fibrillation, n (%)	47 (69.12)	141 (64.68)	49 (80.33)	5.417	0.067
Fibrinolytic therapy, n (%)	24 (35.29)	51 (23.39)	16 (26.23)	3.794	0.150
**ACS classification**					
STEMI, n (%)	22 (32.35)	61 (27.98)	25 (40.98)	3.818	0.148
NSTEMI, n (%)	37 (54.41)	95 (43.58)	22 (36.07)	4.537	0.103
UA, n (%)	22 (32.35)	53 (24.31)	10 (16.39)	4.439	0.109
**Infarct-related artery**					
Left main coronary artery, n (%)	10 (14.71)	37 (16.97)	4 (6.56)	4.124	0.127
Anterior descending, n (%)	40 (58.82)	126 (57.80)	31 (50.82)	2.098	0.925a
Circumflex artery, n (%)	9 (13.24)	30 (13.76)	6 (9.84)	2.031	0.732 a
Right coronary artery, n (%)	23 (33.82)	64 (29.36)	16 (26.23)	7.045	0.491a
**Drug information**					
Dual antiplatelet (aspirin + clopidogrel), n (%)	65 (95.59)	206 (94.50)	57 (93.44)	0.287	0.866b
Oral anticoagulant, n (%)	47 (69.12)	141 (64.68)	49 (80.33)	5.417	0.067
Statin, n (%)	67 (98.53)	216 (99.08)	60 (98.36)	1.008	0.630a
ACEI/ARB, n (%)	54 (79.41)	170 (77.98)	52 (85.25)	1.546	0.462b
β-blocker, n (%)	62 (91.18)	191 (87.61)	54 (88.52)	0.645	0.724b
MACE, n (%)	1 (1.47)	1 (0.46)	0 (0)	1.580	0.606a

a: fisher's precise test; b: chi-square; c: variance analysis.

*ACS* acute coronary syndrome, *STEMI* acute ST-segment elevation myocardial infarction, *NSTEMI* non-acute ST-segment elevation myocardial infarction, *UA* unstable angina pectoris, *ACEI* angiotensin converting enzyme inhibitor, *ARB* angiotensin receptor blocker, *MACE* major adverse cardiovascular event.

Patients who received high dose tirofiban featured a younger age, male gender, a lower mean body mass index, shorter duration time, although no statistical significance was found in these indicators. Meanwhile, the patients reported a similar history of gastrointestinal bleeding, hematencephalon, hypertension, and diabetes mellitus. Among them, no significant difference was found in CYP2C19 polymorphism and the combined use of ticagrelor and aspirin.

### Clinical outcomes

30 days after PCI, 2 cases of MACE events occurred in the 347 patients, including 1 patient in Group L with ischemia-driven target vessel revascularization and 1 patient with nonfatal cerebral hemorrhage in Group M, which showed no statistical difference among the groups (*p* > 0.05). Moreover, no nonfatal myocardial infarction or stent thrombosis was reported in any of the three groups.

In terms of laboratory indicators, paired-sample T test was applied to compare platelet count and functional indexes before and after PCI in Tables 2 and 3. No obvious differences in platelet count were found before and after PCI in Group L (*p* = 0.126) (Table 2), which suggests that medium and high doses of tirofiban could significantly reduce the number of platelets.

**Table 2:** Comparison of platelet count among groups.

	Group L (N = 68)	Group M (N = 218)	Group H (N = 61)	F	*P*
Pre-operation (*10^9/L)	218.72 ± 68.19	226.89 ± 60.44	236.77 ± 77.73	1.230	0.294
Post-operation (*10^9/L)	214.47 ± 70.29	204.85 ± 60.99	209.72 ± 102.57	0.499	0.608
t	1.549	8.540	3.125		
*P*	0.126	< 0.001	0.003

**Table 3:** Comparison of PIR.

	AA%				ADP%				MA_ADP_			
	Pre-operation	Post-operation	t	*p*	Pre-operation	Post-operation	t	*p*	Pre-operation	Post-operation	t	*p*
Group L (N = 68)	70.14 ± 20.78	79.25 ± 19.14	− 6.515	< 0.001	59.58 ± 21.46	77.28 ± 18.05	− 9.737	< 0.001	37.49 ± 11.54	23.52 ± 10.73	12.724	< 0.001
Group M (N = 218)	76.47 ± 20.09	85.82 ± 16.81*	− 9.766	< 0.001	61.39 ± 23.06	84.64 ± 15.52*	− 18.989	< 0.001	34.64 ± 13.40	18.37 ± 10.38*	20.458	< 0.001
Group H (N = 61)	74.88 ± 23.92	87.4 ± 17.11*	− 4.706	< 0.001	63.95 ± 24.94	87.03 ± 16.32*	− 9.709	< 0.001	32.39 ± 14.84	16.27 ± 11.55*	10.791	< 0.001
F	2.369	4.550			0.582	7.003			2.406	8.497		
*P*	0.095	0.011			0.559	0.001			0.092	< 0.001		

*Comparison with low dose group, *p* < 0.05; PIR: platelet inhibition rate.

In order to further observe the effect of tirofiban on platelet function, the platelet inhibition rate of each group before and after operation was comprehensively compared. As shown in Table 3, before the operation, differences in AA%, ADP% and MA_ADP_ were not significant among three groups. After the operation, in all the treatment groups, AA% and ADP% were apparently increased (*p* < 0.001) and MA_ADP_ sharply declined (*p* < 0.001). In addition, Group M and Group H, when compared with Group L, reported markedly improved inhibitory rate of AA% and ADP% (*p* < 0.05) and markedly lower MA_ADP_ after PCI. Further least significant difference T test (LSD-T) analysis revealed no distinct difference trend between Group M and Group H.

### Safety endpoint

In terms of all types of postoperative hemorrhage that indicate tirofiban safety in Table 4, the incidence of total hemorrhage and petechial ecchymosis was much higher in Group H than in the other groups (*p* < 0.005). However, the incidence rate of serious life-threatening bleeding (including massive gastrointestinal bleeding and cerebral hemorrhage) was basically the same among the three groups.

**Table 4:** Comparison of bleeding after operation among three groups.

	Group L (N = 68)	Group M (N = 218)	Group H (N = 61)	χ^2^	*P*
Bleeding gums, n (%)	3 (4.41)	15 (6.88)	5 (8.20)	0.796	0.708b
Gastrointestinal hemorrhage, n (%)	1 (1.47)	3 (1.38)	3 (4.92)	2.908	0.156a
Cerebral hemorrhage, n (%)	0 (0)	1 (0.46)	0 (0)	1.093	1.000a
Hematuria, n (%)	1 (1.47)	2 (0.92)	3 (4.92)	3.992	0.067a
Skin mucosa petechiae, n (%)	2 (2.94)	15 (6.88)	10 (16.39)*	8.772	0.012b
Total haemorrhagic cases, n (%)	7 (10.29)	36 (16.51)	21 (34.43)	13.901	0.001

a: fisher's precise test; b: chi-square.

*Comparison with low dose group, *p* < 0.05.

### Predictors of bleeding by ordinal logistic regression analysis

Multifactor ordinal regression was performed for each kind of postoperative bleeding rate (Table 5). Gastrointestinal hemorrhage and mucosa petechiae after PCI were statistically significant with the OR value of independent risk factor at 4.948 and 2.416, respectively (*p* < 0.05 for both), which indicates that the risk of gastrointestinal bleeding and mucosa petechiae may parallel with the increasing tirofiban dose. A similar incidence rate of cerebral hemorrhage, gingival bleeding and hematuria was observed after PCI (*p* > 0.05).

**Table 5:** Predictors of bleeding by logistic regression analysis.

	β-coefficient	SE	Wald	*P*-value	95% CI		OR
					Lower limit	Upper limit	
Bleeding gums	− 0.012	0.476	0.001	0.979	− 0.945	0.920	0.988
Gastrointestinal hemorrhage	1.599	0.804	3.954	0.047	0.023	3.175	4.948
Cerebral hemorrhage	0.085	2.168	0.002	0.969	− 4.165	4.334	1.089
Hematuria	1.008	0.889	1.286	0.257	− 0.734	2.751	2.740
Skin mucosa petechiae	0.882	0.436	4.097	0.043	0.028	1.735	2.416

Value assignment: Group L = 1, Group M = 2, Group H = 3; Other indicators: No = 0,yes = 1.

## Discussion

Accumulative medical evidence lends support to early coronary interventional therapy for ACS patients^13^. On the premise of safety, the intensity of antithrombotic therapy may reduce the adverse events during peri-operation^13,14,18,24^.

**Figure 1:**
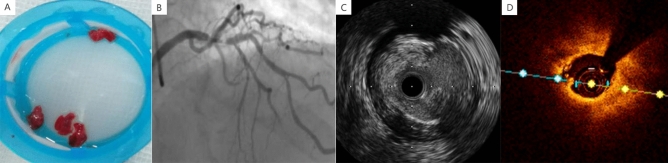
Four different states of thrombus, which were labeled as (**A**–**D**) in turn: (**A**) In vitro; (**B**) Coronary angiography; (**C**) Intravascular ultrasound; (**D**) Optical tomography.

As a non-peptide GPIIb/IIIa antagonist, tirofiban can prevent the binding of fibrinogen to platelet GPIIb/IIIa receptors in order to stop the platelet aggregation and ACS development, thus effectively reducing the risks of thrombosis formation and shedding, distal embolism, no-reflow, and sudden death. Therefore, tirofiban has been recommended by relevant guidelines as a powerful anti-platelet drug that acts on the ultimate anti-platelet pathway if the thrombotic complications occur^5^. The images of in vitro thrombosis, angiography, and intracavity in patients with high thrombotic load were presented from A to D in Fig. 1.

Studies have shown that the tirofiban treatment for high-risk NSTE ACS during the peri-intervention period is safe and effective, which increases the TIMI flow and tissue perfusion, and reduces postoperative complications^25^. Another early study, through the application of tirofiban to the patients with NSTE-ACS, showed that the incidence of adverse cardiovascular events and complications in the tirofiban group was significantly lower than that of the conventional aspirin group^26^, including no distal vascular blockage or recurrent stenocardia, low rate of intra-operative no-reflow and low recurrent rate of myocardial infarction.

Recent studies demonstrate that the upstream tirofiban therapy can significantly improve myocardial perfusion, ST segment resolution, in-hospital mortality rate, and in-hospital sudden cardiac death in patients with STEMI and those with no increased risk of major bleeding^27^. A meta-analysis of randomized trials draws a similar conclusion^28^ that an early routine use of tirofiban, together with the dual antiplatelet therapy with aspirin and clopidrogel, in patients with acute ST-elevation myocardial infarction who have received primary PCI may reduce MACE events without increasing major bleeding rates. Some authors maintain that tirofiban-conferred low target vessel revascularization and stent thrombosis may contribute to the lower rate of the composite of death, recurrent MI, urgent TVR, and bleeding^29^. However, the treatment with GPI has been reported to produce no effect on the composite end point at 1 year in certain trials, reflecting the lack of effect on restenosis^30,31^.

In addition to its own powerful antiplatelet effect, tirofiban may also promote the therapeutic effects of oral antiplatelet drugs. As we all know, aspirin plus ticagrelor is recommended as the first-line anti-platelet agent for ACS patients, but in some specific contexts (anticoagulation for atrial fibrillation, fibrinolytic therapy or the high bleeding risks such as thrombocytopenia, liver disease, or renal disease), aspirin combined with clopidogrel has gained more guideline support^15,18^. For patients taking clopidogrel, insufficient blood concentration is evident at the early stage of drug administration. Even though novel P2Y12 receptor antagonists have recently been shown to enhance the antiplatelet strength in a short time^32^, high residual platelet reactivity (HRPR) has been reported up to 4 or 6 h after the loading dose of P2Y12 receptor in STEMI patients during the primary PCI. As an important supplement to oral medicine, intravenous aggrastat has been proven to provide a more rapid and sustained inhibition of platelet function and bridge the initial treatment gap^33,34^. Clopidogrel, with a less efficacy than new P2Y12 receptor inhibitor^35^, requires the addition of GPI when the blood concentration does not reach the standard level to avoid the formation of stent thrombosis. A recent large retrospective study suggests that a short regimen of GPI is protective against stent thrombosis risk in morphine-treated STEMI patients, who are potentially exposed to increased risk of acute stent thrombosis due to delayed absorption of oral P2Y 12 inhibitors^36^.

In this study, researchers focused on the efficacy and safety of tirofiban at different doses in ACS patients receiving PCI. Only 2 people reported MACEs 30 days later, including 1 case of ischemia-driven target vessel revascularization in Group L and 1 case of postoperative hematencephalon in Group M; no adverse event was found in Group H. Analyses revealed no significant difference in MACE events among three groups (*p* = 0.606), which indicates that postprocedural administration of high-dose tirofiban, combined with traditional dual-antiplatelet treatment, is not superior in decreasing clinical adverse events. The results suggest that compared with the high-dose group, a low dose of tirofiban can achieve similar clinical effects, consistent with a previous study^37^. In terms of laboratory indicators, tirofiban did not significantly reduce platelet counts after PCI in Group L but produced the opposite result in patients receiving medium dose and above. In the comparison of thromboelastic diagram representing platelet function, the postoperative AA% and ADP% were significantly higher than those before the operation, while MA_ADP_ was apparently reduced after PCI, regardless of the dose level of tirofiban. Of note, compared with Group L after the operation, Group M and Group H reported markedly increased inhibitory rates of AA% and ADP% and much lower MA_ADP_. However, no significant difference was evident between Group M and Group H. The findings indicate that tirofiban can synchronously enhance the effect on the three indicators of platelet function, although the inhibition of platelet function is more obvious at medium and high doses than at low doses.

We are also concerned about the safety of Tirofiban. The low rate of major and minor bleeding in the FINESSE study (Facilitated Intervention with Enhanced reperfusion Speed to Stop Events) was found in patients who received GPI in the catheterization laboratory^38^. During the perioperative Intervention in our study, a significantly lower incidence of overall hemorrhage and petechiae was found in the low and medium-dose groups than in the high dose group. However, the incidence of severe bleeding (such as the gastrointestinal massive tract hemorrhage, cerebral hemorrhage) was nearly identical among the three groups. Further logistic regression analysis revealed that gastrointestinal hemorrhage and mucosa petechiae were two independent risk factors (4.948 and 2.416, respectively). Different statistical methods, such as chi-square test and logistics regression, showed that low-dose tirofiban was advantageous in reducing total bleeding and gastrointestinal hemorrhage.

### Study limitations

Several limitations are still present in the current study. First, this study retrospectively probed into the clinical data of patients who received tirofiban during the perioperative period. Among these cases, the majority of patients (94.5%) were treated with aspirin plus clopidogrel. Therefore, the conclusions of this study cannot be generalized to assess general ACS patients with oral aspirin plus ticagrelor. Second, the data analyzed were obtained from a single-center. It remains to be verified whether the derived findings can be generalized for a broad population of ACS patients. Third, the possibility of unspecified or uncorrected factors remained. Despite the relatively large study cohort, it was insufficient to assess infrequent endpoints such as ischemia-driven target vessel revascularization and stent thrombosis. In addition, the duration of observation and follow-up was only 30 days. All of these reasons may result in a low number of MACE events (0.6%) and reduce the reliability of clinical outcomes. Furthermore, an interventional approach may interfere with the rate of bleeding. The radial approach was adopted for the majority of our patients. Therefore, cautions should be taken when generalizing our findings with patients who are treated predominantly by the femoral approach. Finally, aspiration thrombectomy was infrequently employed in our study population. However, the use of thrombectomy devices during STEMI remains controversial^39^.

## Conclusions

The present study shows that the periprocedural administration of low dose tirofiban can provide a satisfactory comprehensive efficacy outcome and lower the overall incidence of bleeding in patients who are pretreated with dual traditional antiplatelet therapy.

## Data Availability

The datasets generated during and/or analysed during the current study are available from the corresponding author on reasonable request.
